# Practical Notes on Diagnosis and Treatment

**Published:** 1907-09-28

**Authors:** 


### PRACTICAL NOTES ON DIAGNOSIS AND TREATMENT.

### Turpentine in Bronchitis.

In my opinion there is no remedy like turpentine in the later stages of bronchitis. But before giving it make sure of the condition of the urine.—*Dr. Vivian Poore.*

### Jaundice in Typhoid Fever.

Jaundice is a very rare complication of typhoid fever. It is usually of the toxæmic form, and seldom depends on obstruction, in the ordinary sense of that term. The prognosis is a very serious one.

### Diabetic Coma.

Success in this condition has followed the use of saline transfusion. A solution of 1 drachm of sodium chloride in a pint of boiled, distilled water may be used, and two or more pints of this at 112° F. may be slowly introduced into the median basilic or other large vein.

### Ruptured Perinæum.

With a tear of the second degree—that is, more than half an inch in extent, it is necessary to put in stitches. The operation is a simple one. All you require is a needle and three pieces of silk-worm gut, preferably stained with methylene blue, because you can so much more easily see to take them out afterwards.—*Dr. H. Spencer.*

### Warts.

The following is a useful application: Salicylic acid, lactic acid, of each 2 parts, collodion 4 parts. Mix. Paint on twice daily.

### Endometritis.

Keep the patient at rest; this is most effectively attained by ordering her to bed. Keep the vagina clean and lessen inflammation in it by antiseptic and sedative or astringent douches; a saturated solution of boric acid if the vagina be sore or the discharge irritating; chloride of zinc ($\frac{1}{4}$ to $\frac{1}{2}$ grain to the ounce) if the discharge is copious. Under such treatment most cases will get well in a week or two.—*Dr. Herman.*

### Tachycardia.

A case is reported by Hausler in which the pulse rate suddenly rose to 200 per minute. The patient was subject to muscular rheumatism. Various remedies, including digitalis, were tried in vain. Then quinine was given in large doses—30 grains within a period of two hours. In the course of a few hours the pulse had fallen to 80. For two years the patient remained in good health. Then the tachycardia returned, but was again successfully treated by large doses of quinine.

